# Supplementary material for: Bacterial DNAemia in Alzheimer’s Disease and Mild Cognitive Impairment: Association with Cognitive Decline, Plasma BDNF Levels, and Inflammatory Response
Source: Int J Mol Sci. 2022 Dec 21;24(1):78. doi: 10.3390/ijms24010078 (PMC9820596; doi:10.3390/ijms24010078)
Supplement: Supplementary file 1 [file ijms-24-00078-s001.zip › ijms-2038007-supplementary.pdf]

*Supplementary Materials*

# Bacterial DNAemia in Alzheimer's Disease and Mild Cognitive Impairment: Association with Cognitive Decline, Plasma BDNF Levels, and Inflammatory Response

**Robertina Giacconi [1,*,†], Patrizia D'Aquila [2,†], Marta Balietti [3], Cinzia Giuli [4], Marco Malavolta [1], Francesco Piacenza [1], Laura Costarelli [5], Demetrio Postacchini [4], Giuseppe Passarino [2], Dina Bellizzi [2,‡] and Mauro Provinciali [1,‡]**

[1] Advanced Technology Center for Aging Research, IRCCS INRCA, 60121 Ancona, Italy
[2] Department of Biology, Ecology and Earth Sciences (DIBEST), University of Calabria, 87036 Rende, Italy
[3] Center for Neurobiology of Aging, IRCCS INRCA, 60121 Ancona, Italy
[4] Geriatrics Operative Unit, IRCCS INRCA, 63900 Fermo, Italy
[5] Clinical Laboratory & Molecular Diagnostics, IRCCS INRCA, 60121 Ancona, Italy
* Correspondence: r.giacconi@inrca.it; Tel.: +39-0718004213; Fax: +39-071206791
† These authors contributed equally to this work and share first authorship.
‡ These authors contributed equally to this work and share last authorship.

**Table S1A. Multivariate linear regression analysis (enter model) for variables independently associated with blood bacterial DNA (BB-DNA) in Alzheimer's disease patients**

| | Unstandardized Coefficients | | Standardized Coefficients | p value |
|---|---|---|---|---|
| | **β** | **Std. Error** | **β** | |
| Sex | 22.979 | 8.864 | 0.322 | 0.012 |
| Age | 0.883 | 0.724 | 0.141 | 0.227 |
| AChEI | 5.502 | 7.567 | 0.082 | 0.470 |
| Benzodiazepines | -9.858 | 10.754 | -0.099 | 0.363 |
| Antidepressants | 10.862 | 9.214 | 0.128 | 0.243 |
| Lipid-lowering medications | -8.041 | 9.812 | -0.092 | 0.415 |
| BDNF plasma levels | 5.776 | 2.571 | 0.239 | **0.028** |
| Alchool consumption | -8.951 | 8.559 | -0.117 | 0.299 |
| Smoking habits | -5.650 | 6.156 | -0.111 | 0.362 |
| PASE | -0.044 | 0.096 | -0.053 | 0.649 |

**AChEI**: Acetylcholinesterase inhibitors; **PASE:** Physical Activity Scale for Elderly. In bolt, significant p value.

**Table S1B. Multivariate linear regression analysis (stepwise backward elimination model) for variables independently associated with blood bacterial DNA (BB-DNA) in Alzheimer's disease patients**

| | Unstandardized Coefficients | | Standardized Coefficients | p value |
|---|---|---|---|---|
| **Model 1** | **β** | **Std. Error** | **β** | |
| Sex | 26.782 | 9.466 | 0.375 | **0.006** |
| Age | 1.042 | 0.738 | 0.166 | 0.163 |
| AChEI | 5.773 | 7.655 | 0.086 | 0.454 |
| Benzodiazepines | -8.024 | 10.999 | -0.080 | 0.468 |
| Antidepressants | 12.769 | 9.887 | 0.151 | 0.201 |
| Lipid lowering medications | -6.769 | 10.109 | -0.078 | 0.506 |
| Smoking | -4.844 | 6.534 | -0.095 | 0.461 |
| Alchool consumption | -8.868 | 8.700 | -0.116 | 0.312 |
| Plasma BDNF levels | 6.046 | 2.889 | 0.250 | **0.040** |
| PASE | -0.081 | 0.104 | -0.099 | 0.438 |
| GDS | -0.855 | 0.623 | -0.164 | 0.175 |
| ADAS-Cog | -0.170 | 0.474 | -0.049 | 0.720 |
| IADL | 1.026 | 2.253 | 0.062 | 0.650 |
| Schooling | 1.088 | 1.208 | 0.108 | 0.371 |
| **Model 2** | | | | |
| Sex | 26.490 | 9.368 | 0.371 | **0.006** |

| | | | | |
|---|---|---|---|---|
| Age | 1.034 | 0.732 | 0.165 | 0.163 |
| AChEI | 6.065 | 7.560 | 0.090 | 0.425 |
| Benzodiazepines | -8.215 | 10.912 | -0.082 | 0.454 |
| Antidepressants | 11.659 | 9.330 | 0.138 | 0.216 |
| Lipid lowering medications | -6.337 | 9.970 | -0.073 | 0.527 |
| Smoking | -5.031 | 6.470 | -0.099 | 0.440 |
| Alchool consumption | -9.035 | 8.629 | -0.118 | 0.299 |
| Plasma BDNF levels | 5.674 | 2.680 | 0.234 | **0.038** |
| PASE | -0.081 | 0.104 | -0.099 | 0.435 |
| GDS | -0.821 | 0.612 | -0.157 | 0.184 |
| IADL | 1.345 | 2.057 | 0.081 | 0.516 |
| Schooling | 1.024 | 1.187 | 0.102 | 0.391 |
| **Model 3** | | | | |
| Sex | 26.523 | 9.325 | 0.371 | **0.006** |
| Age | 1.171 | 0.697 | 0.187 | 0.098 |
| AChEI | 5.859 | 7.519 | 0.087 | 0.439 |
| Benzodiazepines | -8.899 | 10.810 | -0.089 | 0.413 |
| Antidepressants | 11.356 | 9.276 | 0.134 | 0.225 |
| Smoking | -4.571 | 6.400 | -0.090 | 0.478 |
| Alchool consumption | -8.908 | 8.588 | -0.117 | 0.303 |
| Plasma BDNF levels | 5.736 | 2.666 | 0.237 | **0.035** |
| PASE | -0.089 | 0.102 | -0.108 | 0.390 |
| GDS | -0.808 | 0.609 | -0.155 | 0.189 |
| IADL | 1.502 | 2.033 | 0.090 | 0.463 |
| Schooling | 1.135 | 1.169 | 0.113 | 0.335 |
| **Model 4** | | | | |
| Sex | 24.136 | 8.674 | 0.338 | **0.007** |
| Age | 1.109 | 0.689 | 0.177 | 0.112 |
| AChEI | 5.565 | 7.480 | 0.083 | 0.460 |
| Benzodiazepines | -8.539 | 10.758 | -0.085 | 0.430 |
| Antidepressants | 11.227 | 9.240 | 0.133 | 0.229 |
| Alchool consumption | -8.387 | 8.525 | -0.110 | 0.329 |
| Plasma BDNF levels | 5.626 | 2.651 | 0.232 | **0.038** |
| PASE | -0.098 | 0.101 | -0.119 | 0.339 |
| GDS | -0.763 | 0.603 | -0.146 | 0.211 |
| IADL | 1.547 | 2.025 | 0.093 | 0.448 |
| Schooling | 1.365 | 1.119 | 0.135 | 0.227 |
| Model 5 | | | | |
| Sex | 22.852 | 8.473 | 0.320 | **0.009** |
| Age | 1.127 | 0.686 | 0.180 | 0.105 |
| Benzodiazepines | -8.296 | 10.718 | -0.083 | 0.442 |
| Antidepressants | 10.883 | 9.198 | 0.129 | 0.241 |
| Alchool consumption | -9.681 | 8.319 | -0.127 | 0.249 |
| Plasma BDNF levels | 5.652 | 2.642 | 0.234 | **0.036** |

| | | | | |
|---|---|---|---|---|
| PASE | -0.079 | 0.098 | -0.096 | 0.423 |
| GDS | -0.749 | 0.601 | -0.143 | 0.217 |
| IADL | 1.525 | 2.018 | 0.092 | 0.453 |
| Schooling | 1.333 | 1.115 | 0.132 | 0.236 |
| Model 6 | | | | |
| Sex | 23.888 | 8.335 | 0.334 | **0.006** |
| Age | 1.079 | 0.681 | 0.172 | 0.118 |
| Benzodiazepines | -7.538 | 10.638 | -0.075 | 0.481 |
| Antidepressants | 11.771 | 9.094 | 0.139 | 0.200 |
| Alchool consumption | -10.209 | 8.264 | -0.134 | 0.221 |
| Plasma BDNF levels | 5.180 | 2.559 | 0.214 | **0.047** |
| PASE | -0.054 | 0.092 | -0.066 | 0.560 |
| GDS | -0.838 | 0.588 | -0.161 | 0.158 |
| Schooling | 1.316 | 1.111 | 0.130 | 0.240 |
| **Model 7** | | | | |
| Sex | 22.917 | 8.130 | 0.321 | **0.006** |
| Age | 1.159 | 0.664 | 0.185 | 0.086 |
| Benzodiazepines | -7.893 | 10.571 | -0.079 | 0.458 |
| Antidepressants | 12.101 | 9.034 | 0.143 | 0.185 |
| Alchool consumption | -10.665 | 8.188 | -0.140 | 0.197 |
| Plasma BDNF levels | 5.096 | 2.543 | 0.211 | **0.049** |
| GDS | -0.784 | 0.578 | -0.150 | 0.179 |
| Schooling | 1.415 | 1.093 | 0.140 | 0.200 |
| **Model 8** | | | | |
| Sex | 22.993 | 8.104 | 0.322 | 0.006 |
| Age | 1.222 | 0.657 | 0.195 | 0.067 |
| Antidepressants | 12.852 | 8.950 | 0.152 | 0.155 |
| Alchool consumption | -10.456 | 8.158 | -0.137 | 0.204 |
| Plasma BDNF levels | 5.070 | 2.535 | 0.210 | **0.048** |
| GDS | -0.848 | 0.569 | -0.162 | 0.141 |
| Schooling | 1.426 | 1.089 | 0.141 | 0.195 |
| **Model 9** | | | | |
| Sex | 24.888 | 8.004 | 0.348 | 0.003 |
| Age | 1.309 | 0.656 | 0.209 | 0.050 |
| Antidepressant | 12.528 | 8.986 | 0.148 | 0.168 |
| Plasma BDNF levels | 5.029 | 2.546 | 0.208 | **0.049** |
| GDS | -0.771 | 0.569 | -0.148 | 0.179 |
| Schooling | 1.500 | 1.093 | 0.149 | 0.174 |
| **Model 10** | | | | |
| Sex | 21.968 | 7.752 | 0.308 | **0.006** |
| Age | 1.263 | 0.659 | 0.201 | 0.059 |
| Antidepressant | 11.425 | 9.001 | 0.135 | 0.208 |
| Plasma BDNF levels | 5.269 | 2.555 | 0.218 | **0.043** |
| Schooling | 1.317 | 1.091 | 0.131 | 0.231 |

| | | | | |
|---|---|---|---|---|
| **Model 11** | | | | |
| Sex | 19.993 | 7.602 | 0.280 | **0.010** |
| Age | 1.204 | 0.659 | 0.192 | 0.072 |
| Antidepressant | 11.118 | 9.025 | 0.131 | 0.222 |
| Plasma BDNF levels | 5.695 | 2.538 | 0.235 | **0.028** |
| **Model 12** | | | | |
| Sex | 21.796 | 7.485 | 0.305 | **0.005** |
| Age | 1.158 | 0.660 | 0.185 | 0.084 |
| Plasma BDNF levels | 5.596 | 2.546 | 0.231 | **0.031** |

AChEI: Acetylcholinesterase inhibitors; PASE: Physical Activity Scale for Elderly. GDS: Geriatric Depression Scale, IADL: instrumental activities of daily living. In bolt, significant p value.

**Table S1C. Multivariate linear regression analysis for variables independently associated with blood bacterial DNA (BB-DNA) in mild cognitive impairment patients**

| | **Unstandardized Coefficients** | | **Standardized Coefficients** | **p value** |
|---|---|---|---|---|
| | **β** | **Std. Error** | **β** | |
| Sex | 2.019 | 8.064 | 0.036 | 0.803 |
| Age | -0.572 | 0.603 | -0.130 | 0.347 |
| Benzodiazepines | -8.558 | 11.838 | -0.094 | 0.472 |
| Antidepressants | -3.598 | 10.017 | -0.048 | 0.721 |
| Lipid-lowering medications | 8.542 | 8.667 | 0.125 | 0.328 |
| BDNF plasma levels | -3.805 | 2.595 | -0.188 | 0.148 |
| Alchool consumption | 1.468 | 7.933 | 0.024 | 0.854 |
| Smoking habits | 1.985 | 6.521 | 0.042 | 0.762 |
| PASE | 0.026 | 0.099 | 0.037 | 0.795 |

**PASE:** Physical Activity Scale for Elderly

**Table S2. Baseline cognitive tests in relation to blood bacterial DNA (BB-DNA) percentiles in mild cognitive impairment (MCI) and Alzheimer's disease (AD) patients**

| TEST | MCI | | p value | AD | | p value |
|---|---|---|---|---|---|---|
| | BB-DNA$_{low}$ | BB-DNA$_{high}$ | | BB-DNA$_{low}$ | BB-DNA$_{high}$ | |
| ADAS-Cog score | NA | NA | NA | 19.02±1.36 | 22.75±1.53 | 0.069 |
| Attentive matrices | 39.2±1.5 | 38.7±1.3 | 0.808 | 28.8±1.3 | 26.6±1.5 | 0.261 |
| Immediate prose recall | 2.8±0.3 | 3.2±0.3 | 0.466 | 2.1±0.3 | 1.7±0.3 | 0.393 |
| Delayed prose recall | 2.9±0.4 | 3.5±0.3 | 0.197 | 1.3±0.2 | 1.3±0.3 | 0.931 |
| Total prose recall | 6.5±0.6 | 7.5±0.6 | 0.237 | 4.3±0.5 | 3.9±0.6 | 0.595 |
| Word pairing | 7.2±0.5 | 7.4±0.5 | 0.834 | 4.7±0.4 | 3.6±0.4 | 0.065 |
| Semantic verbal fluency | 1.77±0.20 | 2.08±0.18 | 0.247 | 1.00±0.16 | 0.89±0.18 | 0.641 |
| Phonemic verbal fluency | 24.4±1.2 | 24.3±1.0 | 0.928 | NA | NA | NA |
| Corsi Supraspan | 5.0±0.1 | 4.8±0.1 | 0.214 | 4.2±0.2 | 3.7±0.2 | 0.073 |
| Forward digit span | 4.55±0.12 | 4.45±0.10 | 0.550 | 4.10±0.12 | 4.03±0.14 | 0.709 |
| Reverse digit span | 2.93±0.12 | 2.58±0.11 | **0.039** | 2.22±0.13 | 1.80±0.15 | **0.042** |
| LSNS | 28.9±0.9 | 30.2±0.8 | 0.075 | 26.3±0.9 | 27.0±1.0 | 0.597 |

**ADAS-Cog**: Alzheimer's Disease Assessment Scale – Cognitive**; MMSE**: Mini Mental State Examination; **LSNS**: Lubben Social Network Scale; **NA**: Not applicable. ANCOVA analysis corrected for age, sex, and years of schooling.
BB-DNA$_{low}$ values below the 50th percentile and BB-DNA$_{high}$ values above the 50th percentile. In bolt, significant p values.

**Table S3a. Plasma cytokine levels at baseline and after 2-year follow-up in elderly controls (EC), mild cognitive impairment (MCI) and Alzheimer's disease (AD) patients**

| | EC | MCI | AD | p value |
|---|---|---|---|---|
| **TNF-α T0** | 1.01±0.42 | 0.99±0.41 | 1.76±0.38 | NS |
| **TNF-α FU** | 0.97±0.40 | 0.97±0.38 | 1.76±0.37 | NS |
| **IL-10 T0** | 1.11±0.29 | 1.08± 0.29 | 1.61± 0.30 | NS |
| **IL-10 FU** | 1.14±0.13 | 1.06±0.13 | 1.36±0.18 | NS |

**T0**: baseline; **FU**: after 2-years of follow up; **NS**: Not significant.

**Table S3b. Plasma cytokine levels at baseline and after 2-year follow-up in elderly controls (EC), mild cognitive impairment (MCI) and Alzheimer's disease (AD) patients stratified by gender**

| | Males | | | Females | | |
|---|---|---|---|---|---|---|
| | **EC** | **MCI** | **AD** | **EC** | **MCI** | **AD** |
| **TNF-α T0** | 1.02±0.60 | 0.94±0.48 | 1.58±0.42 | 1.07±0.54 | 0.98±0.56 | 1.81±0.53 |
| **TNF-α FU** | 0.91±1.04 | 0.93±0.86 | 2.14±0.81 | 1.05±0.41 | 0.96±0.42 | 1.54±0.41 |
| **IL-10 T0** | 1.28±0.09* | 0.99±0.08 | 0.96±0.08 | 1.05±0.39 | 1.12±0.41 | 1.86±0.42 |
| **IL-10 FU** | 1.16±0.15 | 1.12±0.11 | 1.01±0.11 | 1.19±0.17 | 1.00±0.16 | 1.46±0.17 |

**T0**: baseline; **FU**: after 2-years of follow up; *p<0.05 as compared males MCI and AD

**Figure S1. Blood bacterial DNA (BB-DNA) in elderly controls (EC), Mild Cognitive Impairment (MCI), and Alzheimer's disease (AD) patients after smoke habit stratification**

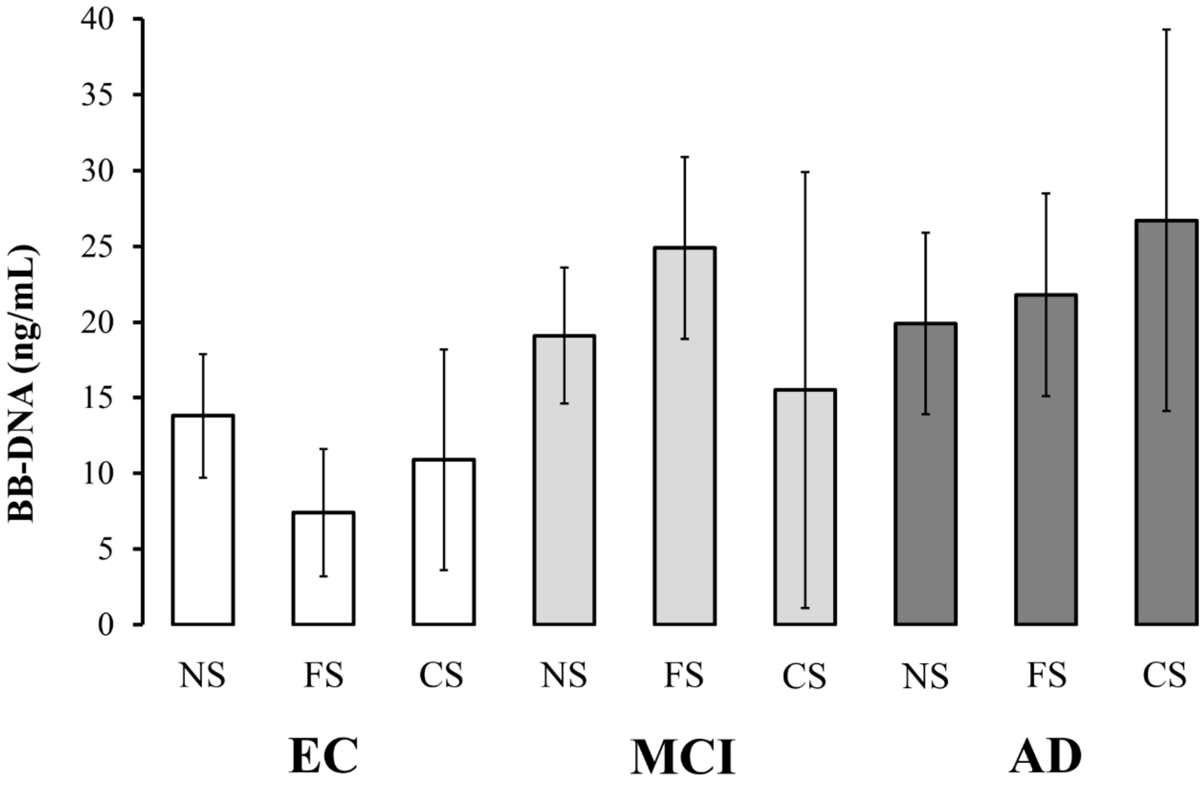


NS, never smoked; FS, former smoker; CS, current smoker. No significant differences were observed in smokers as compared to never smokers in any class of subjects

**Figure S2. BDNF changes after two-year follow-up in relation to blood bacterial DNA (BB-DNA tertiles) in elderly controls (EC), Mild Cognitive Impairment (MCI), and Alzheimer's disease (AD) patients**

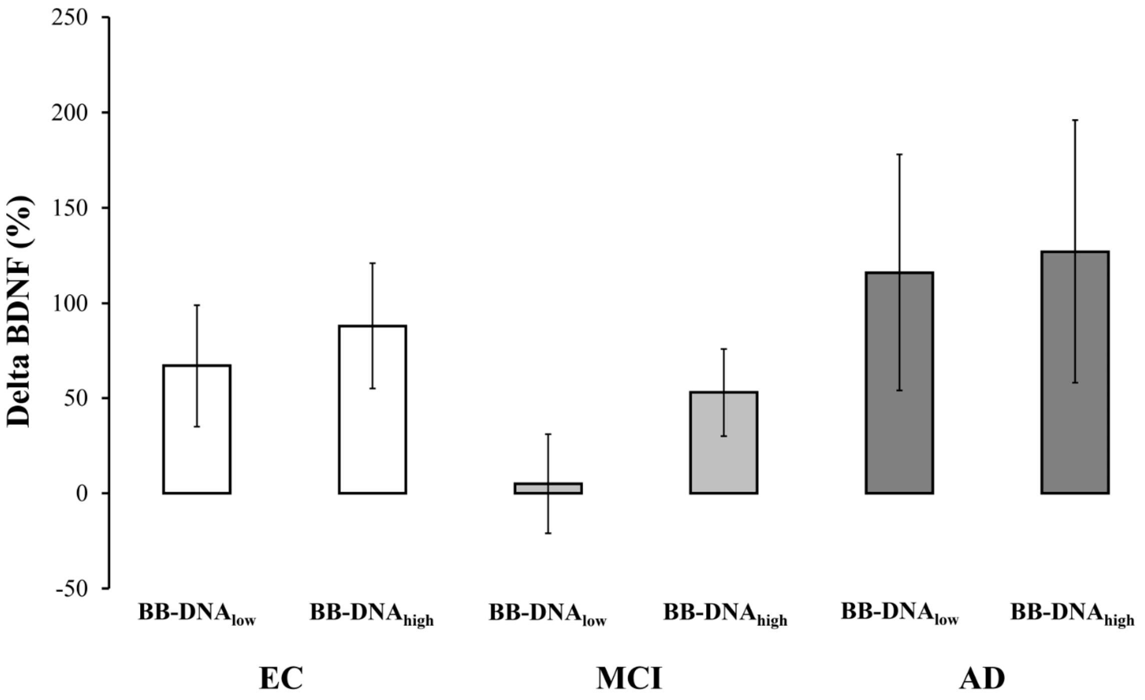


BDNF changes was defined as follows: [(BDNF at follow-up - BDNF at baseline)/ADAS-cog of baseline) x 100]. No significant changes of plasma BDNF levels were found in relation to BB-DNA tertiles in any class of subjects.

**Table S4. Spearman's correlations between blood bacterial DNA (BB-DNA) and plasma cytokine levels at baseline and after 2-years of follow-up in elderly controls (EC), Mild Cognitive Impairment (MCI), and Alzheimer's disease (AD) patients**

| | EC | | MCI | | AD | |
|---|---|---|---|---|---|---|
| | ***r*** | **p value** | ***r*** | **p value** | ***r*** | **p value** |
| **TNF-α T0** | 0.210 | 0.124 | 0.270* | **0.046** | 0.078 | 0.537 |
| **TNF-α FU** | 0.156 | 0.275 | 0.277* | **0.047** | -0.042 | 0.757 |
| **IL-10 T0** | 0.047 | 0.746 | 0.299* | **0.039** | 0.066 | 0.665 |
| **IL-10 FU** | -0.192 | 0.197 | -0.068 | 0.648 | 0.003 | 0.982 |

**T0**: baseline; **FU**: after 2-years of follow up. In bolt, significant p values.
